# Workplace contact patterns in England during the COVID-19 pandemic: Analysis of the Virus Watch prospective cohort study

**DOI:** 10.1016/j.lanepe.2022.100352

**Published:** 2022-04-22

**Authors:** Sarah Beale, Susan Hoskins, Thomas Byrne, Wing Lam Erica Fong, Ellen Fragaszy, Cyril Geismar, Jana Kovar, Annalan M.D. Navaratnam, Vincent Nguyen, Parth Patel, Alexei Yavlinsky, Anne M. Johnson, Martie Van Tongeren, Robert W. Aldridge, Andrew Hayward, Virus Watch Collaborative

**Affiliations:** a Centre for Public Health Data Science, Institute of Health Informatics, University College London, NW1 2DA, UK; b Institute of Epidemiology and Health Care, University College London, London WC1E 7HB, UK; c Department of Infectious Disease Epidemiology, London School of Hygiene and Tropical Medicine, Keppel Street, London WC1E 7HT, UK; d Institute for Global Health, University College London, London WC1N 1EH, UK; e Centre for Occupational and Environmental Health, University of Manchester, Manchester M13 9PL, UK

**Keywords:** COVID-19, Occupation, Epidemiology, Public health

## Abstract

**Background:**

Workplaces are an important potential source of SARS-CoV-2 exposure; however, investigation into workplace contact patterns is lacking. This study aimed to investigate how workplace attendance and features of contact varied between occupations across the COVID-19 pandemic in England.

**Methods:**

Data were obtained from electronic contact diaries (November 2020-November 2021) submitted by employed/self-employed prospective cohort study participants (*n*=4,616). We used mixed models to investigate the effects of occupation and time for: workplace attendance, number of people sharing workspace, time spent sharing workspace, number of close contacts, and usage of face coverings.

**Findings:**

Workplace attendance and contact patterns varied across occupations and time. The predicted probability of intense space sharing during the day was highest for healthcare (78% [95% CI: 75–81%]) and education workers (64% [59%–69%]), who also had the highest probabilities for larger numbers of close contacts (36% [32%–40%] and 38% [33%–43%] respectively). Education workers also demonstrated relatively low predicted probability (51% [44%–57%]) of wearing a face covering during close contact. Across all occupational groups, workspace sharing and close contact increased and usage of face coverings decreased during phases of less stringent restrictions.

**Interpretation:**

Major variations in workplace contact patterns and mask use likely contribute to differential COVID-19 risk. Patterns of variation by occupation and restriction phase may inform interventions for future waves of COVID-19 or other respiratory epidemics. Across occupations, increasing workplace contact and reduced face covering usage is concerning given ongoing high levels of community transmission and emergence of variants.

**Funding:**

Medical Research Council; HM Government; Wellcome Trust

**Research in context**

**Evidence before this study**

To identify studies describing contact patterns by occupation, we searched Medline, Embase, and MedRxiv (up to 05/01/2021) for published research articles or pre-prints with the following search terms: (“occupation” OR “workplace” OR “work-related”) AND (“contact pattern” OR “mixing pattern”). Observational studies describing contact patterns by occupation based on contact surveys or mobile data were included. We identified one relevant USA-based study reporting that workers in retail, accommodation and food service, transportation, healthcare, and manufacturing occupations had the highest mean numbers of daily contacts at work based on surveys conducted between August-December 2020 and March-April 2021.

**Added value of this study**

Using contact diaries collected as part of a large prospective cohort study, we quantified workplace contact patterns by occupation and over time during the second and third waves of the COVID-19 pandemic in England. Contact diaries were completed longitudinally to represent key phases of changing restrictions. We found considerable variation in workplace attendance and features of workplace contact by occupation and over time. Healthcare and education workers experienced more intense workspace sharing and more close contact at work than many other occupational groups, with education workers also less likely to wear a face covering during close contact in the workplace. Across all occupations, workspace sharing and close contact increased and reported use of face coverings decreased during periods of less stringent restrictions, with these trends particularly pronounced in November 2021.

**Implications of all the available evidence**

Workplace contact patterns vary across occupational groups and time. Differences in the frequency and intensity of direct and indirect contact at work are likely to contribute to differential infection risk across occupations. Increases in workspace sharing and close contact and reduced use of face coverings were apparent across all occupations during periods of less intense restrictions, including during periods of high SARS-CoV-2 community transmission.

Alt-text: Unlabelled box

## Background

Severe Acute Respiratory Syndrome Coronavirus 2 (SARS-CoV-2) – the causative agent of the COVID-19 pandemic – spreads through populations via direct or indirect contact between individuals.^1^ Consequently, public health regulations aimed at reducing contact rates overall (e.g., ‘lockdowns’ and sectoral closures) and reducing effective contact where unavoidable (e.g., social distancing, face coverings) have been a cornerstone of the pandemic response worldwide.

Pandemic-related public health interventions have necessarily led to widespread and ongoing changes in the nature of work-related activities. Across several global regions, unprecedented numbers of workers who formerly attended in-person workplaces switched primarily or entirely to working from home or, where not possible to do so, relied on furlough schemes during extended periods of workplace closures.^2^, ^3^, ^4^ Workers in frontline roles have had to adapt to rapidly shifting mitigations, impacted by our evolving understanding of SARS-CoV-2 transmission, the underlying political and material context, and industry-related considerations.^4^, ^5^, ^6^, ^7^ Balancing reopening workplaces with managing ongoing community transmission and the risk of SARS-CoV-2 variants presents an ongoing challenge. Occupation is consequently a fundamental and changing determinant of activity patterns for many people in the working age population, with consequent transmission-relevant implications for others with whom they interact within and outside of work.

Available contact surveys support the important role of work in determining contact rates across the pandemic. Despite large overall reductions in contacts across all settings during the initial months of the pandemic, surveys in the USA and UK suggested that work remained a persistent source of contacts in adults with a less dramatic decrease in contact rates than other locations.^8^, ^9^, ^10^, ^11^ A USA-based survey found that workers in retail, accommodation and food service, transportation, healthcare and manufacturing occupations tended to report the highest mean numbers of daily contacts at work during the COVID-19 pandemic.^8^ In the UK, adults who attended work during the pandemic had substantially higher mean contact rates than those who did not attend their workplace, with this pattern consistent but less pronounced across lockdown periods.^10^ While this initial evidence supports the key influence of work on contact patterns and consequently on potential SARS-CoV-2 exposure, investigation into indirect contact and mitigation in the workplace is also warranted to more thoroughly understand contact in this setting. Furthermore, investigation into features of workplace contact patterns across different occupations is limited and these patterns are likely to vary substantially and influence risk.

The impact of pandemic-related interventions on different sectors, as well as pre-existing differences between occupations, likely influence the degree and routes of work-related SARS-CoV-2 exposure. Occupational differences in risk of infection, morbidity and mortality have emerged in both official statistics and research data from a variety of global regions,^12^, ^13^, ^14^, ^15^, ^16^, ^17^, ^18^, ^19^, ^20^ with patient or public-facing occupations and those requiring in-person attendance tending to demonstrate greater risk of infection and severe outcomes. The contribution of work-related exposure to differential risk is, however, difficult to measure and to delineate from non-work-related factors. Preliminary evidence from the UK suggests that contact at work partially mediates occupational differences in infection risk.^12^^,^^21^ However, understanding the mechanisms underlying differential infection risk by occupation is limited by a lack of data on how specific features of indirect and direct contact differ between occupations – i.e., in their frequency, duration, intensity, and mitigation in different workplace settings. As well as facilitating understanding of differential infection risk, investigation into differential features of occupational contact is relevant to inform both modelling of work-related transmission and to tailor public health interventions for specific occupational contexts during future waves of COVID-19 or other respiratory outbreaks.

The current study aimed to address this gap by quantifying workplace contact patterns by occupation across the second and third waves of the COVID-19 pandemic in England. Using electronic contact diaries completed as part of the Virus Watch cohort study^22^ between November 2020 and November 2021, we set out to investigate how in-person workplace attendance and features of workplace contact, including intensity and duration of space sharing, direct contact, and wearing of face coverings, changed over time and differed between occupational groups.

## Methods

### Ethics approval

The Virus Watch study was approved by the Hampstead NHS Health Research Authority Ethics Committee: 20/HRA/2320, and conformed to the ethical standards set out in the Declaration of Helsinki. All participants provided informed consent for all aspects of the study.

### Participants

Participants in the current study were an adult sub-cohort of the Virus Watch longitudinal cohort study enrolled prior to 09/11/2021 (*n*=50,759). The Virus Watch study recruited whole households using study using social media, SMS, and personalised postal recruitment campaigns supported by general practices. Households that met the following inclusion criteria and where all household members provided consent or assent for participation were eligible: resident in England or Wales, size between 1 and 6 people (due to limitations on survey infrastructure), access to the internet and to an email address, and at least one household member able to complete English-language surveys. Further detail of the main Virus Watch cohort study can be obtained from the study protocol.^22^

Participants were included in the present study if they were:

1. an adult ≥16 years,
2. resident in England, for consistent timing of restrictions and sample size
3. were employed or self-employed full-time or part-time time and reported their occupation upon study registration,
4. and completed at least one contact diary survey between November 2020 and November 2021.

### Exposure

Participants’ entered their occupation as free text upon study registration, which we then assigned UK Standard Occupational Classification (SOC) 2020^23^ codes using semi-automatic processing in Cascot Version 5.6.3.^24^ Where participants listed multiple occupations, the first listed occupation was entered. Occupations were subsequently classified into the following groups based on SOC codes (Supplementary Table S1; see^12^ for further details of occupational classification): administrative and secretarial occupations; healthcare occupations; indoor trade, process & plant occupations; leisure and personal service occupations; managers, directors, and senior officials; outdoor trade occupations; sales and customer service occupations; social care and community protective services; teaching education and childcare occupations; transport and mobile machine operatives; and other professional and associate occupations (broadly office-based, non-essential professional occupations).

### Outcomes

All outcomes for this study were derived from electronic contact diaries delivered using REDCap,^25^ which prompted participants to select all settings where they spent time during a recent 24 h period (between 5am on Monday and 5am Tuesday of the survey week). Participants were prompted to complete the diaries on Wednesday of the survey week, apart from June and November 2021 when they were prompted on Tuesday of the survey week. Workplace attendance was binary coded (yes/no) if they indicated attending their workplace outside of the home. Participants who attended work then responded to the following contact-related items, which were coded as follows in the current study to maximise group size while retaining theoretical significance:

• Maximum number of non-household members with whom the space was shared (regardless of distance): 0, 1–5, 6+ for work
• *If space was shared:* Total amount of time spent sharing space (work/transport) with others: <1 h, 1–4 h, 4+ h for work
• *If space was shared:* Total number of close contacts at work (face-to-face contact within 1 m, spending more than 15 min within 2 m): 0, 1–5, 6+ close contacts
• *If any close contacts:* Frequency of wearing a face covering during close contact: binary for always wear (yes/no)

Diaries corresponded to the following dates, which reflected varying periods of legislation in England: 30 November 2020 (during second English national lockdown), 15 March 2021 (during third English national lockdown), 19 April 2021 (after restrictions on outdoor gatherings and non-essential retail relaxed), 24 May and 28 June 2021 (after restriction on indoor gatherings relaxed), 26 July 2021 (after most remaining COVID-19 restrictions relaxed), 29 September 2021 (after return to school and several months after relaxation of restrictions), and 23 November 2021 (further period after removal of restrictions and prior to concerns about transmission of the Omicron variant).^5^, ^6^, ^7^

### Covariates

Where required (see Statistical Analyses), models were adjusted for the following covariates: age (<30, 30–39, 40–49, 50–59, 60+ years), sex at birth, employment status (full-time or part-time/other), shielding status (recommended to shield vs not), and vaccination status (unvaccinated, 1 dose, 2 doses, 3 doses). Categories of vaccination status were determined based on the UK national vaccination programme (please see https://www.gov.uk/government/collections/covid-19-vaccination-programme), which employed vaccinations in which a two-dose course was considered ‘full vaccination’ with a subsequent third ‘booster’ dose rolled out from September 2021 onwards. Employment status and vaccination status were entered as time-varying covariates.

### Statistical analyses

We used logistic mixed models to investigate the effect of occupational group and time (diary month) on all contact-related outcomes (binomial logistic for workplace attendance and face covering usage; ordinal logistic for maximum number of people in shared workspace, time sharing workspace, and close contacts). Logistic mixed models are able to account for non-independence due to multiple submissions within individuals via the inclusion of a random term, while modelling both ordinal and binary outcomes. To account for possible interactions between occupational group and time, we compared model fit before and after addition of an interaction term using likelihood ratio tests, with interactions included where *p*<0.05 for the likelihood ratio test.

In all models, the most prevalent occupational group - ‘Other professional and associate’ (see Results Table 1) – was set as the reference category. Following the UK Office for National Statistics,^21^ occupation-related results (main effects and interactions) were expressed as predictive probabilities rather than odds ratios to facilitate interpretation of differences between all occupational groups rather than comparison to the reference category.

**Table 1:** Demographic features of study participants.

	*N* = 4616^1^
Occupation	
Administrative & Secretarial	607 (13%)
Healthcare	298 (6.5%)
Indoor Trades, Process & Plant	375 (8.1%)
Leisure & Personal Service	244 (5.3%)
Managers, Directors & Senior Officials	342 (7.4%)
Other professional & associate	1542 (33%)
Outdoor Trades	132 (2.9%)
Sales & Customer Service	255 (5.5%)
Social Care & Community Protective Services	256 (5.5%)
Teaching, Education & Childcare	443 (9.6%)
Transport & Mobile Machine	122 (2.6%)
Age	
<30	457 (9.9%)
30-39	652 (14%)
40-49	670 (15%)
50-59	1423 (31%)
60+	1414 (31%)
Sex	
Female	2347 (51%)
Male	2261 (49%)
Unknown	8 (0.2%)
Ethnicity	
White British	3833 (83%)
White Irish	70 (1.5%)
White Other	384 (8.3%)
South Asian	105 (2.3%)
Other Asian	54 (1.2%)
Black	56 (1.2%)
Mixed	78 (1.7%)
Other Ethnicity	21 (0.5%)
Unknown	15 (0.3%)
Region	
East Midlands	369 (8.0%)
East of England	906 (20%)
London	902 (20%)
North East	247 (5.4%)
North West	498 (11%)
South East	902 (20%)
South West	302 (6.5%)
West Midlands	218 (4.7%)
Yorkshire and The Humber	219 (4.7%)
Unknown	53 (1.1%)
Employment Type	
Full Time	3254 (71%)
Part Time/Other	1362 (29%)
Shielding^2^	252 (8.4%)

^1^*n* (%); ^2^ guidance to stay at home and avoid close contact for people deemed extremely clinically vulnerable to COVID-19, including home working or, if not possible, income support. During study period, relevance workplace guidance remained in place up to the April 2021 survey. See https://www.gov.uk/government/publications/guidance-on-shielding-and-protecting-extremely-vulnerable-persons-from-covid-19

The March 2021 survey, which had the highest response rate and comprised the survey period with the most stringent pandemic-related restrictions, was set as the reference category against which to compare various other time periods of restrictions. Time-related results expressed as odds ratios to facilitate comparison between different phases of restrictions.

Models were adjusted to account for plausible confounders of the relationship under investigation based on the VanderWeele principle of confounder selection.^26^ Consequently, the model for workplace attendance by occupation was adjusted for age, sex, employment status, shielding due to medical vulnerability to severe COVID-19, and vaccination status. While some select occupational groups (e.g., frontline healthcare workers) were targeted for vaccination in the UK, vaccination status was adjusted based on our confounder selection principles as it appears to be associated with occupation more broadly beyond the relatively few groups targeted^27^ and may plausibly influence public activities, including in-person workplace attendance, post-vaccination through mechanisms such as altering perceptions of risk. Adjusted and unadjusted estimates are presented. Other sociodemographic factors were assumed to influence workplace attendance via their relationship with occupation and/or the factors controlled above, and consequently were not included in the model. Models for other features of workplace contact were limited to participants who attended work, and consequently these models were not adjusted as the effects of covariates on these factors were presumed to be mediated through occupation and workplace attendance.

Missing data were sparse across included covariates (Table 1) and we performed complete case analysis.

## Results

**Figure 1:**
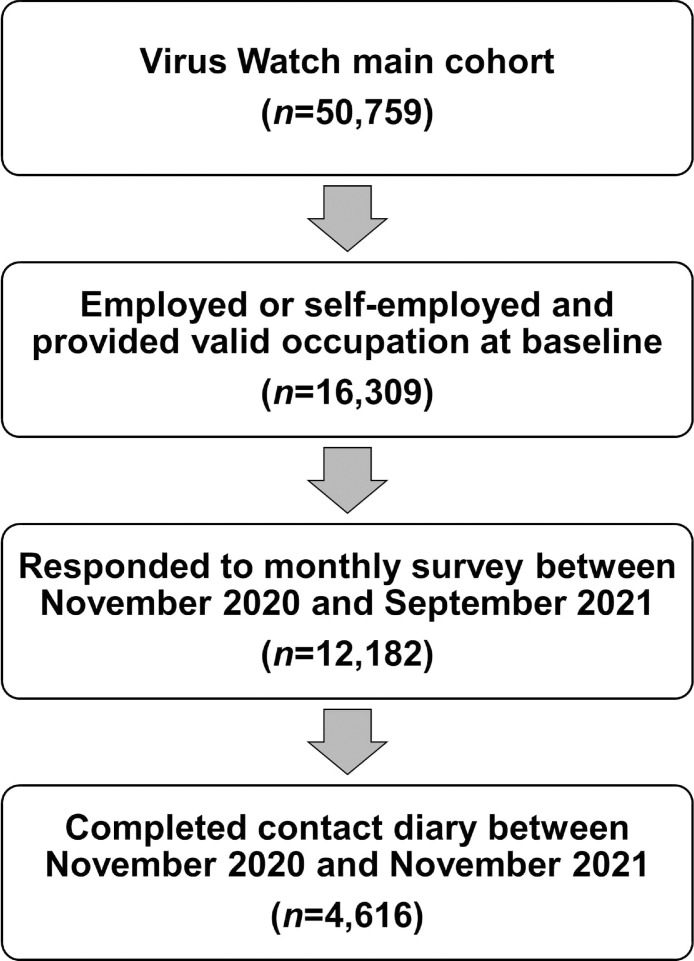
Flow diagram of participant selection.

Participants’ demographic features are reported in Table 1; participant selection is illustrated in Figure 1. A total of 4,616 participants submitted 23,762 contact surveys across the study period, with the number of participants and survey entries for specific items reported in Supplementary Figure S1. Please refer to Supplementary Table S2 for frequencies regarding the number of survey responses per participant. Vaccination status over time is illustrated in Supplementary Table S3.

**Figure 2:**
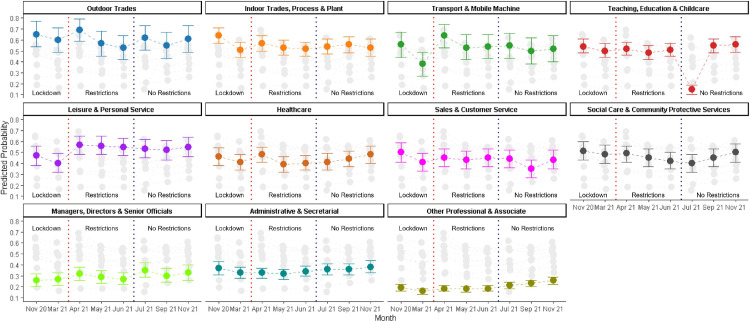
Interaction plot (Occupational Group X Time) for predicted probability of workplace attendance on survey day. Coloured dots illustrate predicted probabilities for each occupational group, with error bars giving 95% confidence intervals; grey background dots indicate predicted probabilities for all other occupational groups.

**Figure 3:**
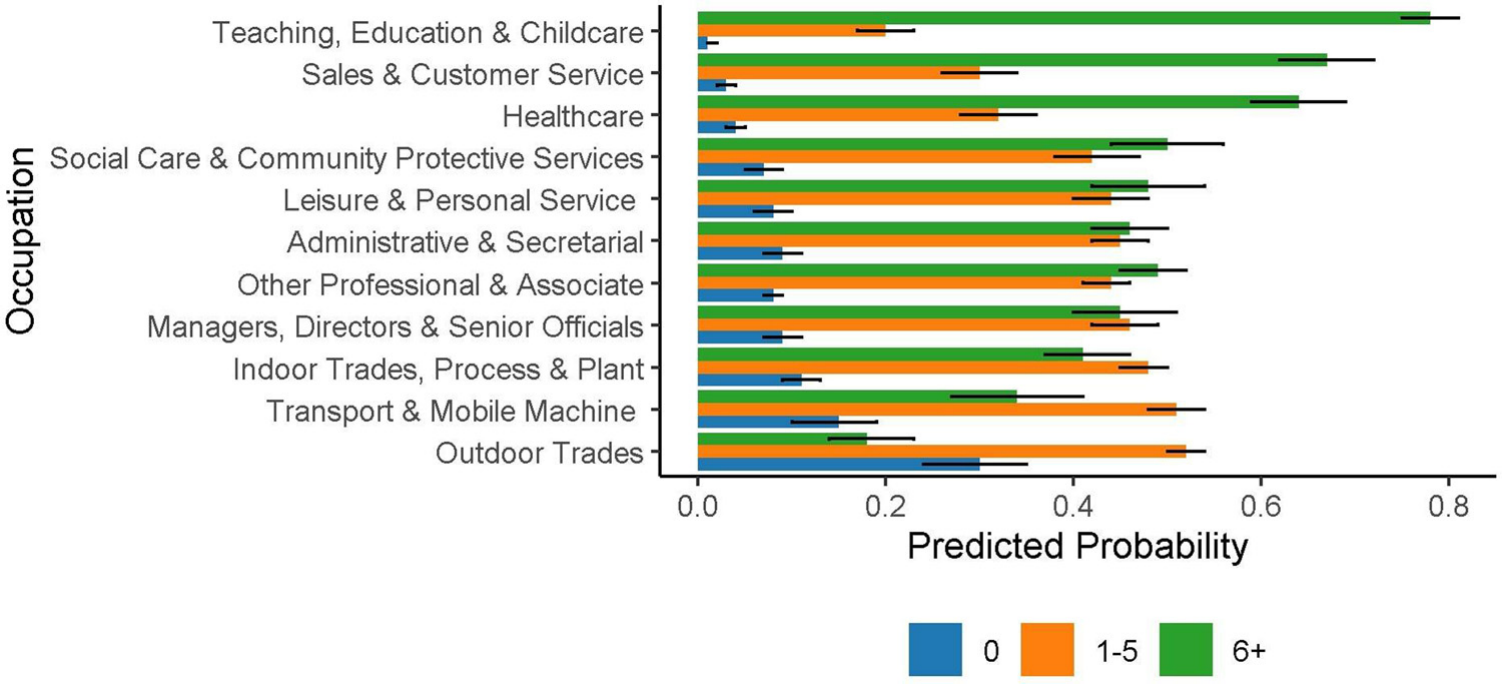
Predicted probabilities with 95% confidence intervals for maximum number of people in workspace by occupational group.

**Figure 4:**
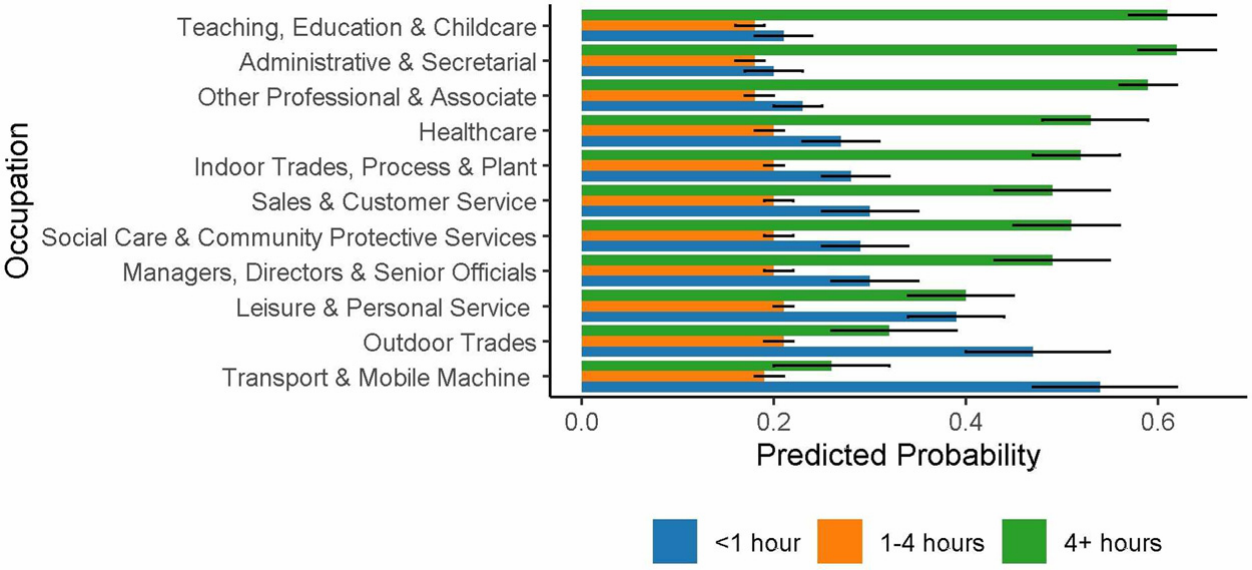
Predicted probabilities with 95% confidence intervals for time spent sharing workspace by occupational group.

**Figure 5:**
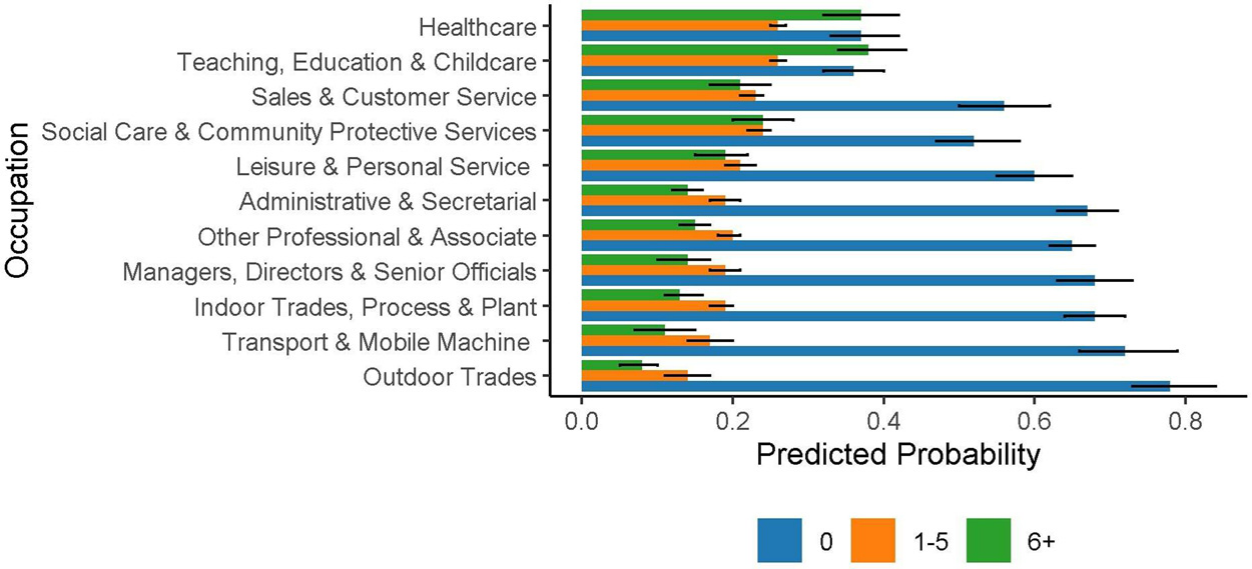
Predicted probabilities with 95% confidence intervals for number of close contacts at work by occupational group.

**Figure 6:**
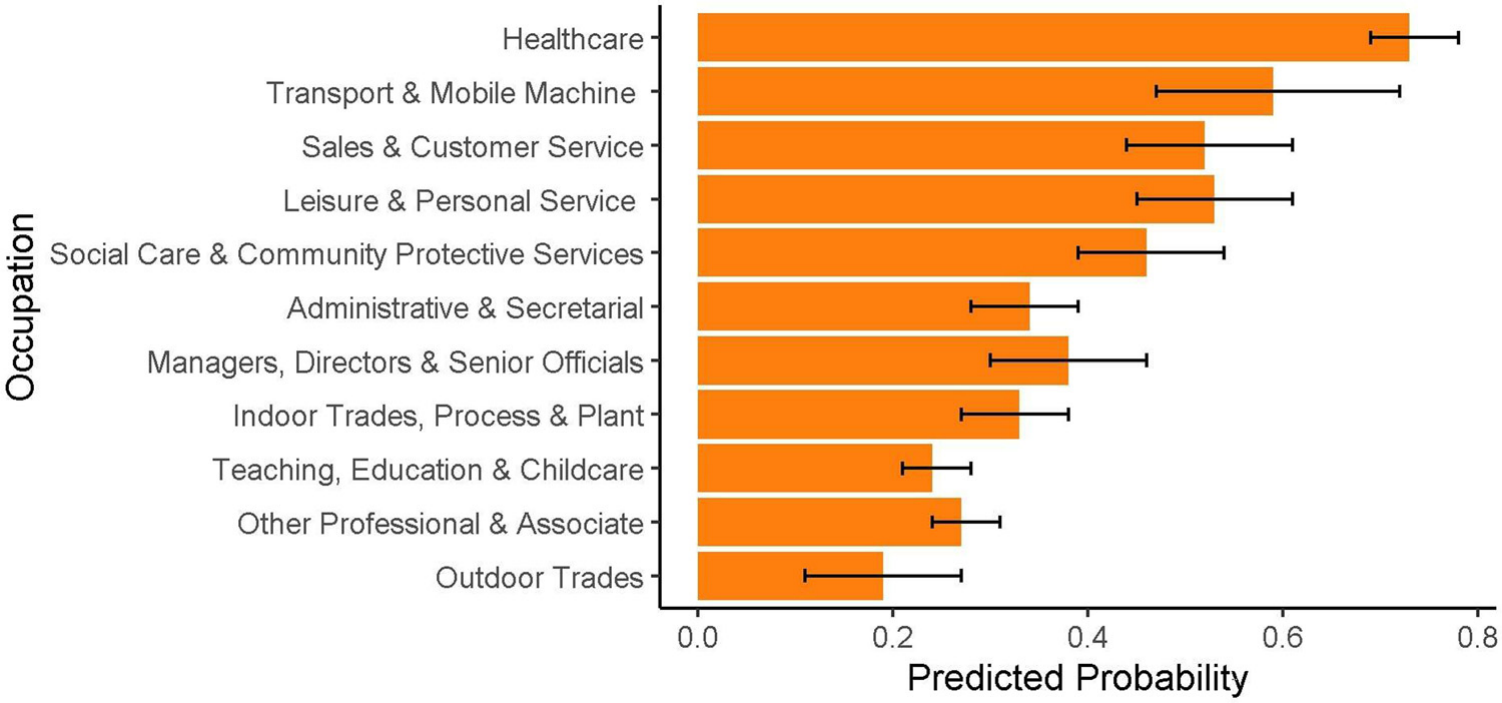
Predicted probabilities with 95% confidence intervals for wearing a face covering during close contact at work by occupational group.

Based on likelihood ratio tests for inclusion of an interaction term between occupational group and time, an interaction was included in the final model for workplace attendance (*p*<0.0001), but not for number of people in the workspace (*p*=0.19), time spent sharing the workspace (*p*=0.20), close contact (*p*=0.25), or use of face coverings during close contact (*p*=0.58). Our mixed effect logistic regression models found that workplace attendance changed differentially over time between occupations, and that the number of people sharing the workspace, time spent sharing the workspace, number of workplace close contacts, and usage of face coverings all varied significantly both by occupation and time period (Figures 2–6).

### Differential probability of workplace attendance over time by occupation

Outdoor Trade occupations persistently exhibited the highest point estimates for predicted likelihood of workplace attendance on the diary day, adjusted for age, sex, employment status, shielding, and vaccination status (Figure 2) (predicted probability = 0.55 [95% confidence interval 0.43,0.67] – 0.69 [0.59,0.79]). Confidence intervals overlapped with Indoor Trade occupations and Transport and Mobile Machine Operatives at all timepoints. For Leisure and Personal Service and Transport occupations, predicted probability of workplace attendance increased between March (respectively 0.40 [0.32, 0.49] and 0.38 [0.27, 0.49]) and April 2021 (respectively 0.57 [0.48, 0.65] and 0.64 [0.53, 0.74]) and remained relatively stable and high afterwards, possibly reflecting effects of sectoral re-openings. Predicted probabilities dropped considerably for Teaching, Education and Childcare occupations in July 2021 (0.15 [0.10, 0.19]) compared to previous and subsequent estimates (0.48 [0.42, 0.55] – 0.56 [0.49, 0.63]), in line with seasonal closures.

Other Professional and Associate occupations had the lowest predicted probability of workplace attendance at all timepoints (0.16 [0.13, 0.18] – 0.26 [0.22, 0.29]), with a trend towards increased in-person attendance from July 2021 onwards. Similar estimates over time across all occupational groups were obtained from the unadjusted model (Supplementary Figure S2).

### Maximum number of people in workspace by occupation and time

The predicted probability of sharing the workspace with more six or more others was highest for Teaching, Education and Childcare occupations (0.78 [0.75, 0.81]), Sales and Customer Service occupations (0.67 [0.62, 0.72]), and Healthcare occupations (0.64 [0.59,0.69]), exceeding estimates for all other occupational groups (Figure 3). Across all occupations, workspace sharing with either 1-5 or 6+ other people was more likely than no sharing at all. Outdoor trade occupations had the highest predicted probability of reporting no workspace sharing (0.30 [0.24,0.35]), exceeding all other groups.

Main effects of time for all workplace contact models are reported in Supplementary Table S4. Compared to March 2021, workers’ odds of more dense workspace sharing across occupational groups were greater in May, June, September and November 2021 (OR range 1.29 [1.03–1.61] in May – 2.13 [1.68–2.69] in November 2021). Confidence intervals for November 2021 indicated elevated odds relative to all other timepoints.

### Time spent sharing workspace by occupation and time

Sharing the workspace for four or more hours was the most likely outcome across most occupational groups (predicted probability range 0.49 [0.43,0.55] – 0.62 [0.58,0.66]), Leisure and personal service, outdoor trade occupations and transport and mobile machine operatives - within which the most common occupations were large goods and delivery drivers (Supplementary Table S1) -had a relatively high likelihood of sharing the workspace with others for less than one hour per day (Figure 4).

The main effect of time indicated increased time sharing the workspace in November 2020 (OR =1.27 [1.02,1.57]) and reduced time sharing the workspace in July and September 2021 (respectively OR =0.77 [0.62,0.96] and 0.99 [0.80,0.93]) compared to March 2021 (Supplementary Table S4).

### Number of close contacts by occupation and time

The predicted probability of having 6 or more contacts across the workday was highest for Teaching, Education and Childcare (0.36 [0.32,0.40]) occupations and Healthcare occupations (0.38 [0.33,0.43]) (Figure 5). For all other occupational groups, the most likely number of close contacts across the workday was zero.

Across occupational groups, workers’ odds of reporting close contacts were greater in November 2020 and between July to November 2021 (OR range 1.39 [1.13–1.72] in November 2020 - 2.19 [1.76–2.72] in November 2021) compared to March 2021. Confidence intervals for November 2021 also indicated elevated odds relative to all other time points except September 2021 (Supplementary Table S4).

### Wearing face covering during close contact by occupation and time

Healthcare occupations had the highest predicted probability of wearing a face covering (0.90 [0.86,0.95]) with confidence intervals for the estimates overlapping only with Transport and Mobile Machine Operatives (0.79 [0.70,0.89]) (Figure 6). There was considerable overlap between estimates for remaining occupations, with Outdoor Trades (0.34 [0.23, 0.45]), Other Professional and Associate occupations (0.51 [0.44,0.57]), and Teaching, Education and Childcare Occupations (0.51 [0.44, 0.57]) demonstrating the lowest predicted probabilities.

All occupations had reduced odds of wearing a face covering during close contact at work in November 2020 and between May to November 2021 (OR range 0.04 [0.02, 0.08]– 0.28 [0.17, 0.48]) relative to March 2021 (Supplementary Table S4).

## Discussion

### Key findings and interpretation

This study aimed to investigate in-person workplace attendance and workplace contact patterns by occupation across the second and third waves of the COVID-19 pandemic in England. Differential patterns of in-person workplace attendance and features of workplace contact were identified across occupational groups, with variation in at-risk groups depending on the characteristic under investigation. Across all occupations, intensity of workspace sharing and close contact increased during periods of less stringent restrictions relative to the third national lockdown and likelihood of wearing a face covering during close contact decreased. These trends were particularly prominent in November 2021, several months after most public health measures – including mandates around social distancing and mask wearing in some public spaces – had been lifted in England despite high ongoing levels of community transmission.^5^^,^^6^

Probability of workplace attendance changed differentially over time between occupational groups, broadly in line with previous classifications for frontline versus non-frontline roles.^28^ Trade and transport occupations demonstrated high probability of in-person attendance, as did leisure/service and teaching occupations in line with periods of sectoral opening. Conversely, Other Professional and Associate occupations - broadly comprising non-essential, office-based roles - persistently demonstrated the lowest likelihood of in-person workplace attendance with a high proportion continuing to work mainly at home even after lifting of restrictions.

Occupational sectors identified in previous studies and surveillance data as having elevated risk of SARS-CoV-2 infection - largely essential and public-facing occupations including healthcare, transportation, education, indoor trade and service occupations^12^, ^13^, ^14^, ^15^, ^16^, ^17^, ^18^, ^19^, ^20^ - also tended to demonstrate high likelihood of workplace attendance and/or multiple elements of workplace contact-related risk in the current study. Notably, workers in teaching/education/childcare occupations demonstrated high probabilities of workplace attendance, workspace sharing and close contact, as well as relatively low probability of wearing a face covering during close contact. While workspace sharing and close contact may be difficult to avoid in many educational roles, uptake of face coverings as well as potential environmental mitigations addressing indirect and direct contact-related risk may be particularly beneficial in these environments. Healthcare workers also tend to demonstrate elevated infection risk based on research and surveillance data, particularly during the first pandemic wave.^20^^,^^29^, ^30^, ^31^, ^32^ In the present study, they also demonstrated more intense space sharing and close contact than most other groups. While it could not be directly assessed in the present study, access to personal protective equipment and other mitigation methods including prioritisation for vaccination was likely instrumental in mitigating contact-related risk in later pandemic waves.

COVID-related mortality is strongly influenced by clinical factors as well as SARS-CoV-2 exposure.^17^ However, occupational groups with several elements of contact-related risk in the present study – such as healthcare workers and elementary trade and service occupations – also broadly reflected those with elevated mortality rates in UK national statistics available disaggregated by occupation up to December 2020.^17^^,^^33^ Teaching occupations demonstrated multiple elements of contact-related risk but not elevated mortality. Given that elevated infection risk has been observed in teachers based on research and surveillance data, clinical and other non-work-related risk factors likely contribute considerably to this discrepancy. Transport occupations, which demonstrated high workplace attendance but relatively low levels of contact-related exposure in the present study, were also a notable exception. The sample of transport workers in the current study was dominated by large goods and delivery drivers, while excess mortality has been observed primarily in public-facing transport occupations. This likely contributes to the observed discrepancy, along with confounding of mortality risk by clinical risk factors. We lacked power to disaggregate occupational groups, including transport, further in the present study.

Despite observed variation in features of workplace contact across occupational groups, workspaces tended to be shared with others for long periods of time across most occupations. Probability of close contact across occupations was lower than that of space sharing, suggesting that sharing tended to be socially distanced. However, reporting may be particularly influenced by social desirability bias - close contact was a target of public health messaging in England during the pandemic - as well as the stringent definition of contact. While overall probabilities of close contact may have consequently been underestimated, this is unlikely to have influenced between-occupational differences. Furthermore, indirect contact can still present a transmission risk, particularly in high-footfall, poorly ventilated indoor environments.^1^^,^^34^ In light of ongoing high levels of SARS-CoV-2 transmission and emergence of new variants, increasing workspace sharing and close contact across occupations with reduced usage of face coverings is likely a major contributor to transmission.

Public health interventions to reduce the number of individuals sharing workspaces - including promoting working from home where possible - and to promote the uptake of mitigation methods such as face coverings are important measures to slow transmission. By describing differential contact patterns by occupation and across time periods comprising different stringency of restrictions, these findings provide indication of work-related mitigations that may be beneficial in the case of future waves of COVID-19 and potentially in future epidemics of other respiratory viruses. However, as all contact surveys were collected in the context of different phases of COVID-19, investigation into workplace contact patterns in a non-pandemic phase are necessary to inform response during the initial emergence of a future outbreak. Workplace attendance and contact patterns may also be influenced by employer-level as well as national-level mandates and guidance, but it was not possible to disaggregate these effects in the present study. Both policy-level changes in restrictions and individual-level adherence to restrictions may influence contact patterns at work. Investigation into the psychosocial processes underpinning behavioural change at work - such as fatigue, frustration, and/or confusion with restrictions- was beyond the scope of this study, but presents an important avenue for further research to inform relevant interventions.

### Strengths and limitations

Strengths of this study include the large, diverse cohort that allowed us to investigate workplace contact across a range of occupational groups. Repeated surveys covered key periods of the second and third pandemic waves in England, and were repeated after major changes in pandemic-related restrictions over time.

Several important limitations, however, should be considered in interpreting these findings. The study cohort is not representative of the English population, with a relatively high concentration of older workers and, likely relatedly, high vaccine uptake. Both occupation and contact patterns were measured in broad categories. Occupational groups are likely to include specific roles with different risk profiles, but we lacked power to investigate in further detail. Notably, contact patterns amongst the Transport and Mobile Machine operative group may have been influenced by the relatively large proportion of large goods and delivery drivers relative to public transport workers; however, we were unable to disaggregate these occupations further. We also lacked power to test potential sociodemographic effect modifiers of the relationship between occupation and workplace contact patterns. The necessary use of broad ordinal scales for some outcomes may have masked granular differences between occupations. Self-reported contact and activities may have been impacted by recall bias and social desirability bias, particularly during periods of stringent restrictions. Findings are not generalisable to the first pandemic wave when many infections may have occurred, particularly in some frontline occupational groups such as health and social care workers.^20^^,^^29^, ^30^, ^31^ Linking these findings directly to infection risk was beyond the scope of the present study – which aimed to provide a granular account of changes in features of workplace contact across the pandemic – due to a lack of sufficient data at all time-periods to facilitate adequately-powered analysis. Broader investigation into the relationship between workplace exposure and infection risk has been conducted elsewhere,^12^ and further investigation into potentially moderators of the relationship – such as environmental mitigations and vaccination status of workers and their contacts – is recommended.

Each contact survey related to a single weekday in order to facilitate detailed recall of activities, but may not have been representative of a normative weekday for participants. Due to survey timing and infrastructure, surveys were generally sent one day after the relevant period (5am Monday – 5am Tuesday) which may have impacted detailed recall. For participants with hybrid or part-time working patterns, the selection of Monday may have influenced the likelihood of in-person workplace attendance as mid-week workplace working may be more common. Due to the survey design, it was not possible to attribute a specific reason for workplace non-attendance, which may have been driven by workplace closure, self-isolation, or other reasons for non-attendance including those independent of the pandemic. Estimates of in-person workplace attendance may have been systematically biased downwards during some timepoints, particularly July 2021 when high levels of community transmission led to a surge in requests to self-isolate via the English National Health Service Test and Trace mobile application^35^; these requests may have affected some public-facing occupations differentially. Further details around the age structure of contacts, the environmental features of workspaces, and mitigation methods used at work were beyond the scope of this survey to limit burden and recall-related issues. In particular, the use of personal protective equipment (PPE), face covering types and details of related behaviour, and estimated air volume and ventilation in indoor spaces could not be assessed, and are relevant moderators of contact-related risk. Further detail around person-hours in close contact and changes in the number of people in shared airspaces throughout the workday would be informative. It was also not possible to attribute the source of close contact or space sharing (i.e., whether this was driven by colleagues and/or the public).

## Conclusions

Our findings provide quantitative evidence of differential workplace attendance and workplace contact patterns by occupation during the COVID-19 pandemic. This study also demonstrates change over time in workplace contact across the pandemic, with evidence of a greater degree of workspace sharing and close contact and lower probability of wearing a face covering during periods of less stringent restrictions, including during periods of high community COVID-19 transmission. These findings provide preliminary evidence around variation in risk-relevant features of workplace contact to inform further research and risk mitigation of SARS-CoV-2 and other respiratory infections in the workplace.

## Declaration of interests

AH serves on the UK New and Emerging Respiratory Virus Threats Advisory Group. AJ and AH are members of the COVID-19 transmission sub-group of the Scientific Advisory Group for Emergencies (SAGE). AJ is Chair of the UK Strategic Coordination of Health of the Public Research board and is a member of COVID National Core studies oversight group.

## Appendix Supplementary materials

Image, application 1
