# Supplementary material for: Workplace contact patterns in England during the COVID-19 pandemic: Analysis of the Virus Watch prospective cohort study
Source: Lancet Reg Health Eur. 2022 Apr 22;16:100352. doi: 10.1016/j.lanepe.2022.100352 (PMC9023315; doi:10.1016/j.lanepe.2022.100352)
Supplement: Supplementary file 1 [file mmc1.pdf]

Supplementary Materials

Occupational Classification

Supplementary Table S1. UK Standard Occupational Classification 2020 (SOC-2020) Codes within Virus Watch Occupational Categories

Virus Watch Occupational Category	UK SOC-2020 Codes	Three Most Prevalent Occupations in Study Sample* (SOC-2020 Unit Group)
Administrative & Secretarial Occupations	4111-4217, 9211, 9219, 9233	1. Other administrative occupations n.e.c. (20%, n= 122)<br>2. Book-keepers, payroll managers, and wage clerks (9.4%, n=57)<br>3. Personal assistants and other secretaries (7.7%, n=47)
Healthcare Occupations	2211-2259, 3211-3219, 3240, 6131-6133	1. Other nursing professionals (19%, n=56)<br>2. Generalist medical practitioners (8.4%, n=25)<br>3. Nursing auxiliaries and assistants (8.1%, n=24)
Indoor Trades, Process & Plant Occupations	5211-5250, 5315-5317, 5321-5323, 5411-5449, 8111-8149, 8160, 9131-9139, 9241-9259	1. Warehouse operatives (10%, n=38)<br>2. Metalworking production and maintenance fitters (8.8%, n=33)<br>3. Electricians and electrical fitters (7.5%, n=28)
Leisure & Personal Service Occupations	1221-1225, 1252, 1253, 1256, 1257, 6121, 6129, 6211-6250, 9221-9229, 9231, 9261-9269	1. Cleaners and domestics (15%, n=37)<br>2. Kitchen and catering assistants (9.8%, n=24)<br>3. Hairdressers and barbers (8.6%, n=21)

Managers, Directors & Senior Officials	1111-1161, 1171,1172, 1211, 1212, 1231, 1241-1243, 1251, 1254, 1255, 1258, 1259	1. Financial managers and directors (15%, n=50)<br>2. Functional managers and directors n.e.c. (10%, n=35)<br>3. Marketing, sales, and advertising directors (10%, n=35)
Other Professionals & Associate Professionals	2111-2162, 2411-2455, 2471-2494, 3111-3133, 3411-3582	1. Programmers and software development professionals (6.5%, n=100)<br>2. Business and financial project management professionals (4.2%, n=64)<br>3. Management consultants and business analysts (3.8%, n=59)
Outdoor Trade Occupations	5111-5119, 5311-5314, 5319, 5330, 8151-8159, 9111- 9129	1. Gardeners and landscape gardeners (21%, n=28)<br>2. Construction and building trades n.e.c. (17%, n=23)<br>3. Construction operatives n.e.c. (12%, n=16)
Sales & Customer Service Occupations	7111-7220	1. Sales and retail assistants (35%, n=88)<br>2. Customer service occupations n.e.c. (11%, n=29)<br>3. Retail cashiers and check-out operators (11%, n=29)
Social Care & Community Protective Services	1162, 1163, 1232, 2461-2469, 3221-3229, 3311-3319, 6134-6138, 6311-6312	1. Care workers and home carers (24%, n=61)<br>2. Welfare and housing associate professionals n.e.c. (12%, n=30)<br>3. Youth and community workers (9%, n=23)
Teaching, Education & Childcare Occupations	1233, 2311-2329, 3231, 3232, 6111-6117, 9232	1. Higher education teaching professionals

		(17%, n=75)<br>2. Education advisers and school inspectors (16%, n=70)<br>3. Secondary education teaching professionals (13%, n=56)
Transport & Mobile Machine Operatives	8211-8239	1. Large goods vehicle drivers (22%, n=27)<br>2. Delivery drivers and couriers (19%, n=23)<br>3. Bus and coach drivers (15%, n=18)

Abbreviations: n.e.c. = not elsewhere classified; * Limited to three most prevalent occupations per category to prevent declarative disclosure and due to large number of occupations across sample (*n*=381)

Supplementary Figure S1. Flow Diagram of Contact Survey Item Responses

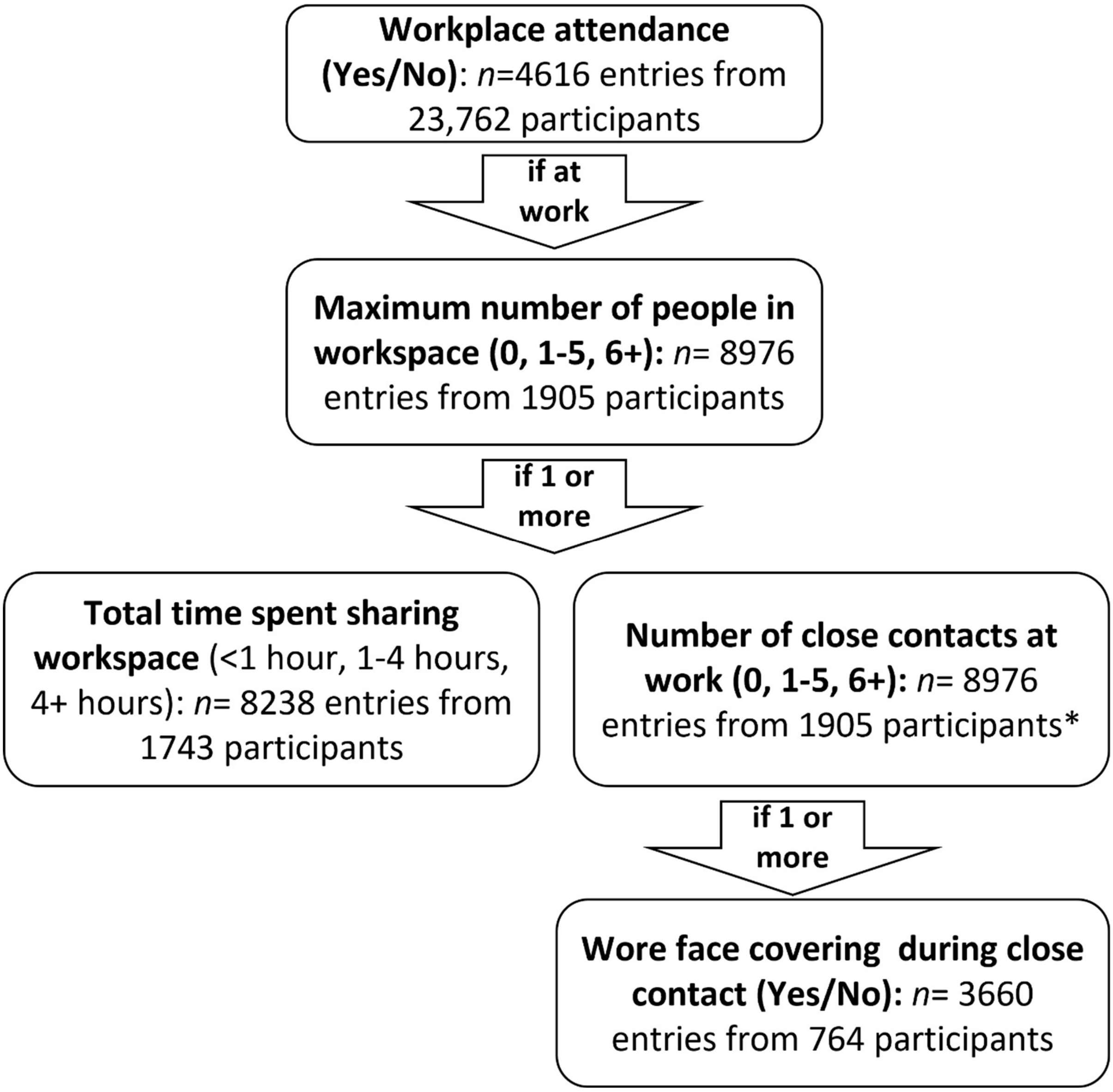

***Note:** Despite survey nesting, models for space sharing and close contact share the same number of participants as the number of close contacts was necessarily 0 if space was not shared. This could not be disaggregated for time spent in contact.

Supplementary Table S2. Number of Contact Surveys Completed by Participants

Number of Survey Responses	Frequency	Percentage
1	591	12.80
2	438	9.49
3	372	8.06
4	389	8.43
5	410	8.88
6	521	11.29
7	713	15.45
8	1182	25.61
Total	4616	100

Supplementary Table S3. Participant Vaccination Status over Time

	**Total –** n (row %)	**Unvaccinated** - n (row %)	**One Dose** – n (row %)	**Two Doses** - n (row %)	**Three Doses** – n (row %)
**November 2020**	3,049 (100%)	3,049 (100%)	0 (0%)	0 (0%)	0 (0%)
**March 2021**	3,397 (100%)	1,440 (42%)	1,859 (55%)	98 (2.9%)	0 (0%)
**April 2021**	3,361 (100%)	795 (24%)	1,938 (58%)	628 (19%)	0 (0%)
**May 2021**	3,144 (100%)	393 (12%)	1,013 (32%)	1,738 (55%)	0 (0%)
**June 2021**	2,989 (100%)	138 (4.6%)	536 (18%)	2,315 (77%)	0 (0%)
**July 2021**	2,863 (100%)	102 (3.6%)	335 (12%)	2,426 (85%)	0 (0%)
**September 2021**	2,538 (100%)	63 (2.5%)	142 (5.6%)	2,259 (89%)	74 (2.9%)
**November 2021**	2,421 (100%)	42 (1.7%)	80 (3.3%)	1,151 (48%)	1,148 (47%)

Supplementary Figure S2. Interaction Plot (Occupational Group X Time) for Predicted Probability of Workplace Attendance on Survey Day (Unadjusted Model). Coloured dots illustrate predicted probabilities for each occupational group, with error bars giving 95% confidence intervals; grey background dots indicate predicted probabilities for all other occupational groups.

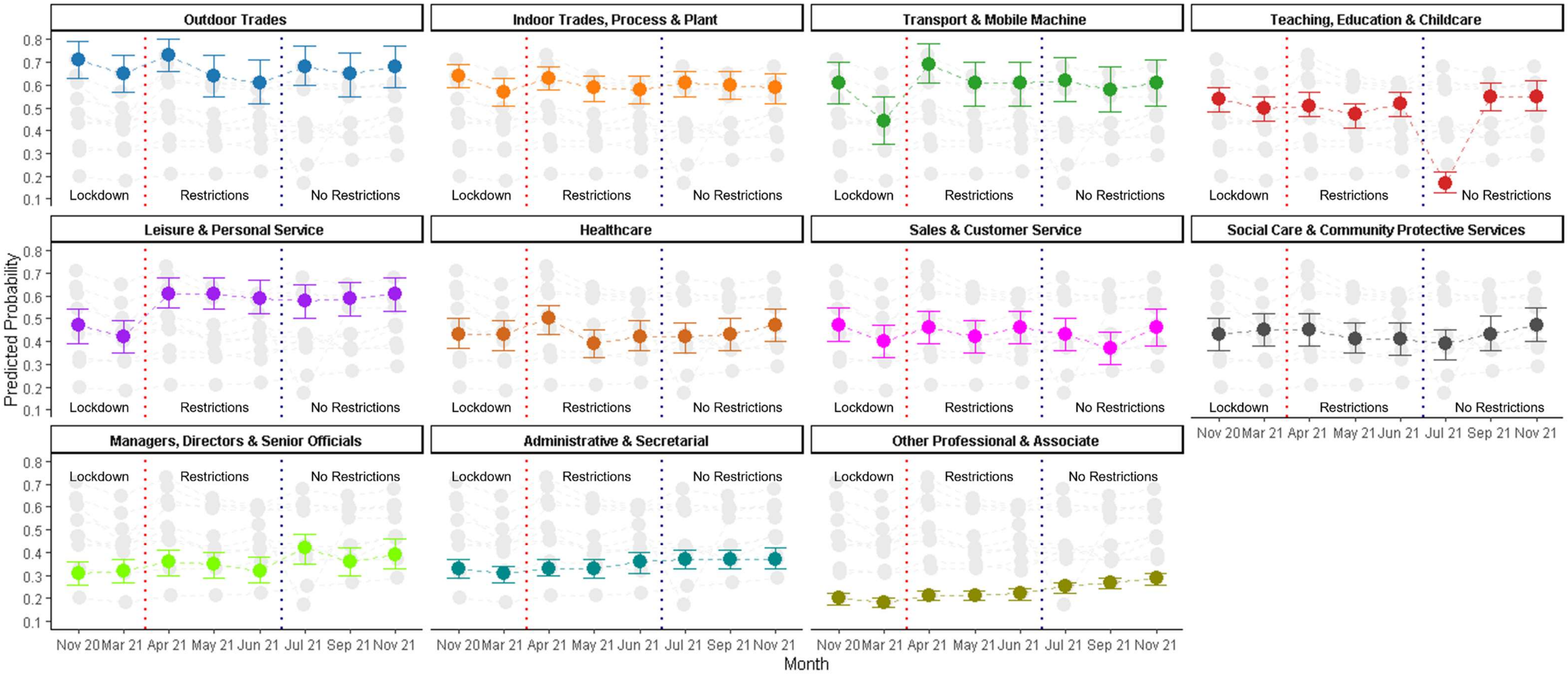

Supplementary Table S4. Main Effects of Time for Workplace Contact Models

	**Number of People in Workspace –** OR (95% CI)	**Time Spent Sharing Workspace –** OR (95% CI)	**Number of Workplace Close Contacts–** OR (95% CI)	**Face Covering During Close Contact –** OR (95% CI)
**November 2020**	1.13 (0.91, 1.41)	1.27 (1.02, 1.57)	1.39 (1.13, 1.72)	0.22 (0.13, 0.37)
**March 2021**	REF	REF	REF	REF
**April 2021**	1.24 (1.00, 1.53)	1.06 (0.86, 1.29)	1.09 (0.89, 1.34)	0.64 (0.39, 1.04)
**May 2021**	1.29 (1.03, 1.61)	0.98 (0.79, 1.20)	1.22 (0.99, 1.51)	0.28 (0.17, 0.48)
**June 2021**	1.33 (1.06, 1.66)	1.02 (0.82, 1.25)	1.16 (0.94, 1.44)	0.13 (0.07, 0.22)
**July 2021**	1.20 (0.95, 1.50)	0.77 (0.62, 0.96)	1.42 (1.14, 1.77)	0.13 (0.07, 0.23)
**September 2021**	1.94 (1.54, 2.44)	0.99 (0.80, 1.23)	1.94 (1.56, 2.42)	0.04 (0.02, 0.08)
**November 2021**	2.13 (1.68, 2.69)	1.13 (0.91, 1.41)	2.19 (1.76, 2.72)	0.05 (0.03, 0.09)

Note: OR = odds ratio; CI = confidence intervals; REF= reference category
